# From scepticism to integration: adoption of mobile health apps in Danish chiropractic practice—a mixed methods study

**DOI:** 10.1186/s12998-025-00604-0

**Published:** 2025-10-24

**Authors:** Mette Mouritsen Sørensen, Madalina Jäger, Mette Jensen Stochkendahl

**Affiliations:** 1 Chiropractic Knowledge Hub, Campusvej 55, 5230 Odense M, Denmark; 2 Department of Sports Science and Clinical Biomechanics, University of Southern Denmark, Campusvej 55, 5230 Odense M, Denmark; 3 Danish Centre for Motivation and Behavior Science (DRIVEN), University of Southern Denmark, Campusvej 55, 5230 Odense M, Denmark

**Keywords:** mHealth, Chiropractic, Musculoskeletal pain, Implementation, Mixed methods, Barriers and facilitators, Self-management tools, Usability, Patient engagement, Primary care settings

## Abstract

**Background:**

Musculoskeletal (MSK) pain affects quality of life and burdens healthcare systems, increasing the need for effective self-management tools. Mobile health apps offer a potential solution, but their adoption in clinical practice remains limited due to concerns about usability, patient engagement, and data security. Denmark is highly digitalized, with various digital health initiatives already in place. However, little is known about chiropractors’ engagement with health apps. This study examines how Danish chiropractors utilize and perceive health apps in their practice, identifying key barriers and facilitators to adoption and providing insights for broader implementation in primary care settings to improve self-management support for patients with MSK pain.

**Methods:**

A two-staged sequential mixed-methods exploratory study was conducted, comprising a quantitative survey distributed to active members of the Danish Chiropractic Association in June 2022 followed by 11 qualitative semi-structured interviews with a purposively sampled subset of respondents, conducted from November 2022 to May 2023. The quantitative data were analysed for frequencies and proportions, while qualitative data were analysed using framework analysis. Integration of both datasets was performed to identify patterns and insights.

**Results:**

Out of 650 chiropractors, 294 (45%) responded to the survey, with 288 included in the analysis. The results indicated that while over half of the chiropractors used health apps personally, only 15% utilized them in patient care. Key barriers included limited knowledge, time constraints, and narrow scope of use, while facilitators included app user-friendliness and positive patient feedback. Qualitative insights revealed varied attitudes towards mHealth, with some chiropractors perceiving apps as valuable tools for patient support, while others expressed scepticism regarding their relevance and effectiveness.

**Conclusion:**

This study provides valuable insights into the current state of health app integration within Danish chiropractic practice, highlighting the need for targeted education and policy changes to facilitate the adoption of digital health tools. It emphasizes the importance of addressing both patient and provider perspectives to enhance self-management strategies for MSK pain through mHealth solutions. The findings contribute to the broader discourse on digital health integration in healthcare, particularly within the chiropractic profession.

**Supplementary Information:**

The online version contains supplementary material available at 10.1186/s12998-025-00604-0.

## Background

Musculoskeletal (MSK) pain affects individuals of all ages, leading to disability, reduced quality of life, and often necessitating rehabilitation [1, 2]. MSK pain places a significant burden on healthcare systems due to increased visits and treatments, as well as on society through sickness absence, early retirement, and reduced productivity [1]. With a substantial number of patients needing rehabilitation and an increasing pressure on healthcare systems, finding ways to guide self-management is imperative. Mobile health (mHealth) potentially offers such guidance for self-management of MSK pain [3, 4]. mHealth is defined as the use of mobile technology, like health apps, to provide patients with healthcare support and healthcare providers (HCP) with technical support [5]. The number of health apps available has rapidly increased, and with most people owning a smartphone, they are easily accessible [6–8], however not all these apps are necessarily evidence-based. Despite the potential of mHealth, integration into the healthcare system has not progressed as expected due to implementation barriers faced by HCPs and patients [9]. In recent years, studies have explored the perceptions of HCPs and their use of health apps in their practice in the primary and secondary care sectors [7, 10–21]. When the apps are validated and backed by evidence [7, 10, 12, 15–17], are easy to use [10, 11, 13, 14, 16, 17, 22], and personalised to the patient’s condition [10, 14, 16] HCPs perceive them as useful and beneficial, and a mean to improve patient compliance with treatment [16, 20]. Health apps are also valued when they provide both the patient and the HCPs access to information [10, 14, 15, 18, 20], facilitate communication [11, 15, 17], or enable sharing of information across digital platforms [11, 12, 16, 17]. Additionally, financial incentives for HCPs to recommend apps can further promote their use [11, 13, 17]. Conversely, apps are not regarded as potential tools for clinical practice when HCPs have a low digital affinity [10, 11, 13, 14, 17] or are unaware of appropriate, effective and validated health apps [7, 13, 19, 20], find that apps are time-consuming to use [7, 10–12, 20], have concerns about data security [11–13, 16–18, 20], or fear that health apps compromise the professional relationship with the patient [11, 13–15, 18].

Denmark is a highly digitalised country [23–25], with the widespread implementation of digital solutions in the healthcare system being a political priority [26]. This includes a centralised, electronic patient health record system that provides access to personal health data for both patient and HCPs in secondary sector records (named *MinSundhed*). Other examples include digital communication between different sectors of the healthcare system, sharing of diagnostic imaging between primary and secondary sector, and health apps provided by Danish health authorities, like Medicinkortet, an app which allows patients to track and renew their prescribed medication. HCPs in the primary care sector also use electronic health records.

The chiropractic profession is one of the three main professions in Danish primary care that manage patients with MSK. Chiropractors are well-integrated in the primary healthcare system, and benefit from the digital resources mentioned as well as state authorisation, publicly funded reimbursement, and formal academic education. Most Danish chiropractors are employed within the primary care sector in private clinics, either as clinic owner or as employees and working as MSK specialists. Patients typically self-refer to a chiropractor, referrals from doctors not being needed, and pay a fee for service [27]. Given the overwhelming number of patients with MSK pain, almost 1 mio with low back pain and more than 0.6 mio with neck pain in Denmark (population of 6 mio) [28], the trend towards digitalisation of healthcare, and the focus on increased self-management for MSK conditions, it is important to understand how MSK providers, including chiropractors, perceive and use health apps. This understanding is crucial for the wider implementation of health apps in primary care to enhance chiropractors' support for patients in self-managing MSK pain. This study aims to explore Danish chiropractors’ utilisation, experiences with, and attitudes towards health apps in the management of patients with musculoskeletal pain.

## Methods

### Overall approach

**Fig. 1:**
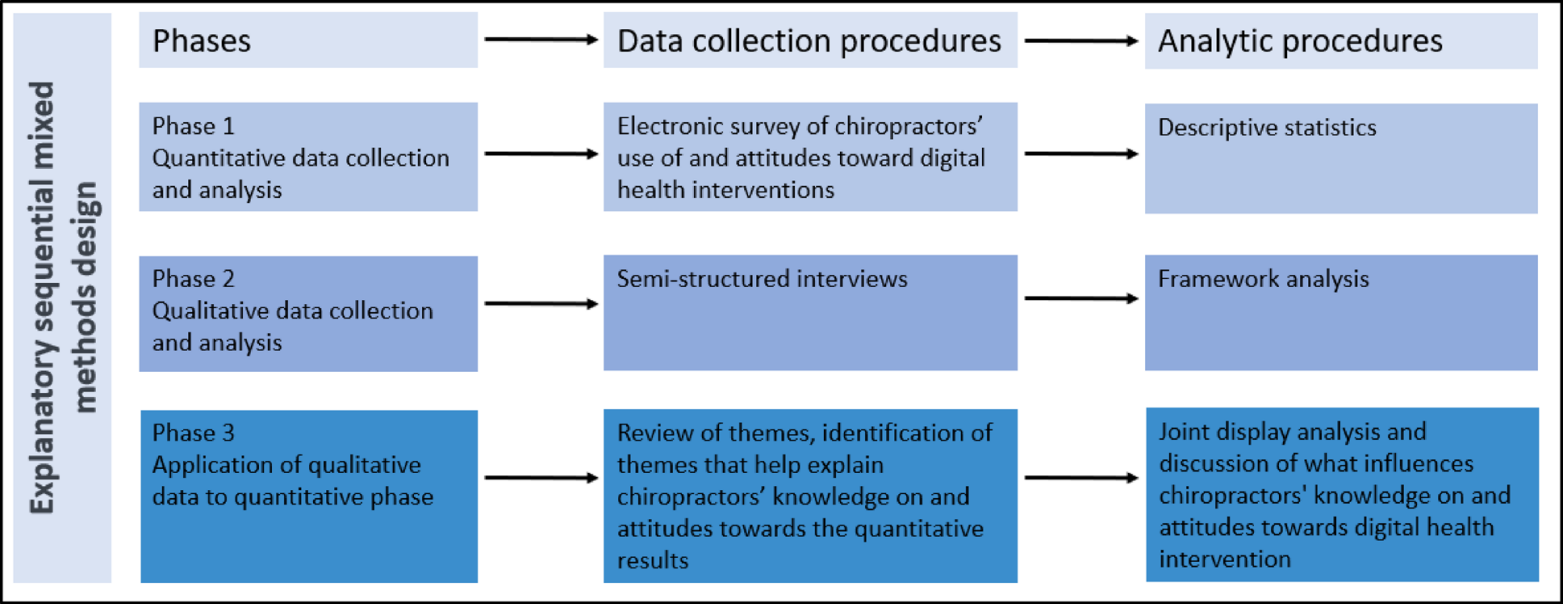
Procedural diagram of the mixed methods research study

We conducted a two-staged explanatory sequential mixed-methods study of Danish primary care chiropractors including a quantitative, cross-sectional survey and a qualitative exploration using semi-structured interviews, followed by an integration of the two datasets (Fig. 1). The reporting of the study will follow the checklist for Mixed Methods Reporting in Rehabilitation & Health Sciences (MMR-RHS) [29].

### Quantitative data

#### Recruitment, participants, and data collection

First, we conducted a cross-sectional study. All active members of the Danish Chiropractic Association (650 chiropractors) received an email invitation in June 2022 with a link to an electronic survey (SurveyXact). The chiropractors were included in the study if they consented to participation and worked in a primary care sector clinic. Reminders were sent by email two, four, and eight weeks later to those who had not completed the questionnaire.

#### Developing the survey

The survey drew on questions from international surveys of primary HCPs’ use of and attitudes towards digital health [7, 12, 16, 19]. The questions were translated into Danish and adapted to fit the Danish setting and chiropractic profession. We duplicated questions to distinguish between personal use and use with patients. When inquiring about which apps the chiropractors used, we added categorical response categories naming commonly used Danish health apps to the free-text response option, and the response option “never” to questions about how often they used apps. Before distribution, the questionnaire was pilot tested by chiropractic students and minor changes were made.

The questionnaire consisted of 34 questions with categorised response options (additional file 1) sorted into four domains: 1) sociodemographic information (i.e. gender, age, experience, type of employment, geographic region, number of patients seen per hour); 2) The chiropractors’ own use of health apps (*yes/no*, types of apps); 3) The chiropractors’ use of health apps with their patients (*yes/no*, types of apps, frequency of use, interest in learning more); and 4) The chiropractors´ perceptions of the acceptability of health apps. In this domain, chiropractors were asked to indicate their level of agreement on a 5-point Likert scale (from *Strongly disagree* to *Strongly agree*) with statements regarding barriers to the implementation of health apps, and advantages for both patients and chiropractors of using health apps. In addition, a free text response option allowed for further comments from the respondents. In the last question, the chiropractors were asked if they would be interested in participating in a follow-up interview, and if so to provide their contact information.

#### Quantitative data analysis

Categorised data were reported as frequencies and proportions. The data analysis was conducted in STATA 18 (StataCorp LLC, College Station, Texas, USA).

### Qualitative data

#### Participants

From the 84 survey respondents who indicated their willingness to participate in an interview, we purposefully sampled chiropractors with various experiences with app use, attitudes, and gender. Based on their gender (female/male) and their answers regarding their own use of health apps (yes/no) and their use with their patients (yes/no), we divided respondents into six groups and used a random list generator to compile a list for each group. The first three chiropractors on each list were invited via email to participate. Reminders were sent two weeks after the invitation. If a respondent declined, the next person on the list was invited. Table 1 shows the characteristics of the participants.

**Table 1:** Characteristics of interview participants

Pseudonym	Age category (years)	Gender	Position	Their own use of health apps	Use health apps with their patients
Anne	41–50	Female	Employed	Yes	No
Bridget	51–60	Female	Other	No	No
Ben	51–60	Male	Owner	Yes	Yes
Emma	31–40	Female	Employed	Yes	Yes
Harry	61–70	Male	Employed	No	No
John	61–70	Male	Owner	Yes	Yes
Camilla	41–50	Female	Owner	Yes	No
Michael	21–30	Male	Employed	No	No
Miles	31–40	Male	Employed	Yes	Yes
Petra	61–70	Female	Employed	Yes	Yes
Simon	21–30	Male	Employed	Yes	No

#### Procedure

Based on the responses in the survey and the Theoretical Framework of Acceptability (TFA) [30], an adaptive interview guide was developed focusing on the chiropractors’ experience with, attitudes towards, and their use and acceptability of digital health interventions. The interview guide was pilot-tested and subsequently revised before the interviews were conducted (Additional file 2).

Eleven semi-structured, individual interviews lasting 33–59 min were conducted in Danish online by MMS using the electronic video platform, Microsoft Teams (version 1.6) from November 2022 to May 2023. The interviews were video recorded by Teams, and the automated inbuild transcription function in Teams was used to make an initial transcription. Subsequently, the transcripts were checked for accuracy by comparing the videos and the transcripts, and any missed or misinterpreted parts were corrected by MMS.

#### Data analysis and theoretical framework

The transcripts were transferred into NVivo software version 1.7.1 for analysis. Data were analysed using Ritchie et al.'s “Framework analysis” with five stages of qualitative analysis: familiarization, identifying a thematic framework (TFA), indexing, sorting, framework summarization, and abstraction and interpretation [31]. All transcripts were coded in Danish by MMS in collaboration with MJS. The analytic process is described more detailed in additional file 3. For this paper, illustrative quotes were translated into English.

The TFA was used to inform the thematic framework. TFA outlines seven constructs—affective attitude, burden, ethicality, intervention coherence, opportunity costs, perceived effectiveness, and self-efficacy—which together capture participants’ cognitive and emotional responses to an intervention. In this study, the TFA constructs guided both the development of initial codes and the interpretation of themes.”

### Integration of quantitative and qualitative data

We used joint display to organise the quantitative and qualitative findings and identify links and patterns between the two sets of data [32, 33]. In the analysis, we mapped quantitative findings onto the themes identified in the interviews to identify areas of convergence and divergence between the datasets, and areas where the qualitative findings expanded the quantitative results. Finally, areas of complementarity, defined as new insights or interpretations based on the integrated findings, were identified.

## Results

### Survey findings

Of the 650 chiropractors invited, 294 (45%) responded to the invitation. Six did not work in the primary care setting, leaving 288 (44.3%) in the study, of which 27 (9.4%) only partially finished the questionnaire. The respondents were mainly female (57%), between 51 and 60 years of age, and clinic owners (Table 2). More than half the chiropractors responding used apps themselves, mainly apps from the Danish health authorities, exercise apps, and fitness tracking apps. Only 15% of the respondents used apps with their patients. Chiropractors using apps themselves used apps with patients more often compared to those not using apps themselves.

**Table 2:** Respondent characteristics and their use of health apps

Demographics n(%)	Using apps with their patients		
	No	Yes	Total
	245 (85.1)	43 (14.9)	288 (100.0)
Gender			
Male	104 (42.4)	15 (34.9)	119 (41.3)
Female	136 (55.5)	28 (65.1)	164 (56.9)
Missing	5 (2.0)	0 (0.0)	5 (1.7)
Age			
21–30 years	62 (25.3)	5 (11.6)	67 (23.3)
31–40 years	35 (14.3)	8 (18.6)	43 (14.9)
41–50 years	64 (26.1)	10 (23.3)	74 (25.7)
51–60 years	62 (25.3)	14 (32.6)	76 (26.4)
> 61 years	20 (8.2)	6 (14.0)	26 (9.0)
Missing	2 (0.8)	0 (0.0)	2 (0.7)
Years since graduation			
< 21 years	157 (64.1)	21 (48.8)	178 (61.8)
> 21 years	83 (33.9)	22 (51.2)	105 (36.5)
Missing	5 (2.0)	0 (0.0)	5 (1.7)
Employment*			
Clinic owner	138 (56.3)	27 (62.8)	165 (57.3)
Employee in clinic	101 (41.2)	15 (34.9)	116 (40.3)
Other employment	19 (7.8)	10 (23.3)	29 (10.1)
Region of practice*			
Eastern Denmark	102 (41.6)	17 (39.5)	119 (41.3)
Western Denmark	145 (59.2)	26 (60.5)	171 (59.4)
Number of patients seen per hour			
1–2	8 (3.3)	1 (2.3)	9 (3.1)
3–4	166 (67.8)	30 (69.8)	196 (68.1)
5–6	58 (23.7)	11 (25.6)	69 (24.0)
> 6	9 (3.7)	1 (2.3)	10 (3.5)
Missing	4 (1.6)	0 (0.0)	4 (1.4)

Chiropractors use of health apps n (%)			
Uses apps themselves			
No	107 (43.7)	8 (18.6)	115 (39.9)
Yes	134 (54.7)	35 (81.4)	169 (58.7)
Missing	4 (1.6)	0 (0.0)	4 (1.4)
Which apps do they use themselves*			
Exercise	64 (26.1)	24 (55.8)	88 (30.6)
Fitness tracking (e.g. step count)	96 (39.2)	22 (51.2)	118 (41.0)
Apps from the Danish health authority (i.e. “*MinSundhed*”, “*Medicinkortet*”)	105 (42.9)	30 (69.8)	135 (46.9)
Sleep	34 (13.9)	8 (18.6)	42 (14.6)
Nutrition and weight	26 (10.6)	6 (14.0)	32 (11.1)
Smoking cessation	0 (0.0)	1 (2.3)	1 (0.3)
Menstrual cycle	25 (10.2)	7 (16.3)	32 (11.1)
Stress and mental health	12 (4.9)	7 (16.3)	19 (6.6)
Other	5 (2.0)	2 (4.7)	7 (2.4)
Which apps do they use with their patients**			
Exercise		34 (79.1)	34 (11.8)
Fitness tracking (e.g. step counts)		14 (32.6)	14 (4.9)
Apps from the Danish health authority (i.e. “*MinSundhed*”, “*Medicinkortet*”)		20 (46.5)	20 (6.9)
Sleep		6 (14.0)	6 (2.1)
Nutrition and weight		4 (9.3)	4 (1.4)
Smoking cessation		1 (2.3)	1 (0.3)
Menstrual cycle		1 (2.3)	1 (0.3)
Stress and mental health		11 (25.6)	11 (3.8)
Other		8 (18.6)	8 (2.8)
How often they recommend health apps to their patients			
Daily		7 (16.3)	7 (2.4)
Weekly		20 (46.5)	20 (6.9)
Monthly		13 (30.2)	13 (4.5)
Rarely		3 (7.0)	3 (1.0)
Missing		0 (0.0)	245 (85.1)
Wanting to learn more about health apps			
No	44 (18.0)	5 (11.6)	49 (17.0)
Yes	174 (71.0)	36 (83.7)	210 (72.9)
Missing	27 (11.0)	2 (4.7)	29 (10.1)

*Multiple responses permitted; percentages sum to > 100%

^**^Only participants using apps with patients received this question

**Fig. 2:**
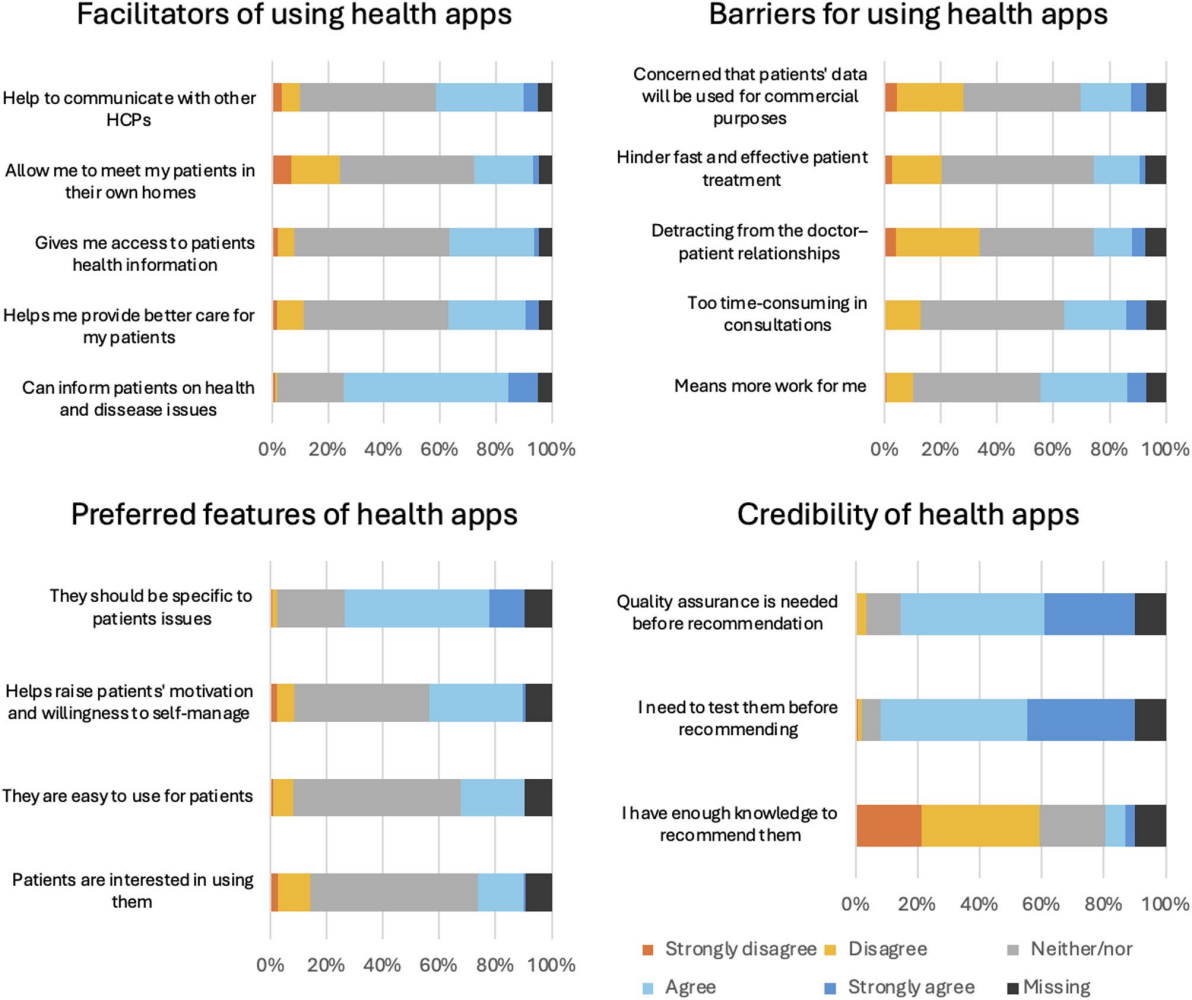
Chiropractors’ perceptions of the acceptability of health apps, n = 288

**Fig. 3:**
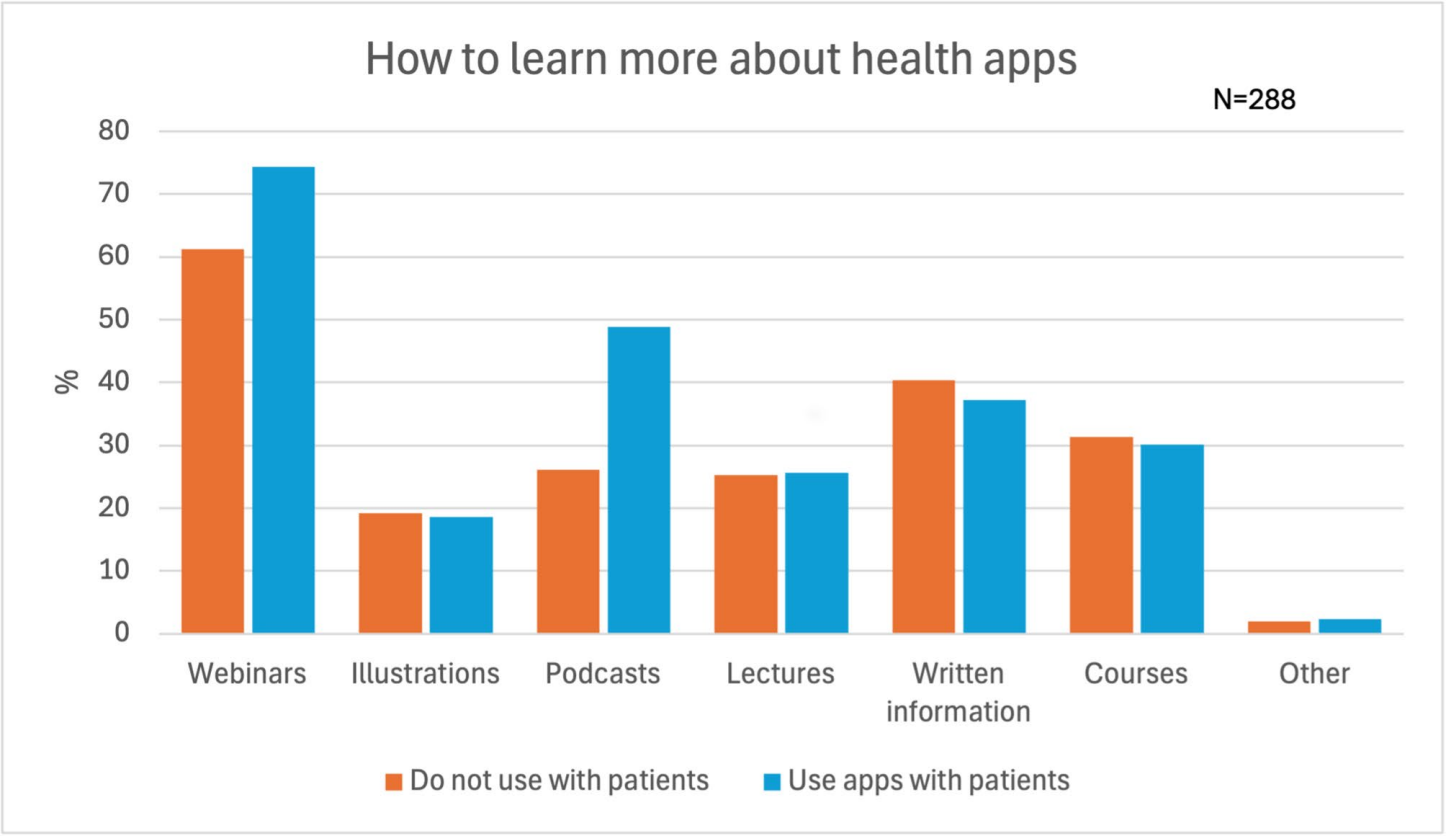
How chiropractors prefer to learn about health apps

The chiropractors’ attitudes towards health apps are presented in Fig. 2. The majority of the chiropractors answered neither/nor to most of the statements except for mostly agreeing that *quality assurance is needed before recommending apps* and disagreeing with *having enough knowledge to recommend apps* (Domain: The chiropractors’ perception of the acceptability of health apps) Further, they agreed that they *need to test the apps before recommending them* (Domain: The chiropractors’ perception of the acceptability of health apps). Preferred channels for learning about health apps were through webinars, written information, and courses (Fig. 3).

### Interview findings

The qualitative results are categorised into two main themes *(1. Aligning the perceived purpose of health apps with clinical practice values; 2. Chiropractors divided: knowledge, barriers, and attitudes shape the usage of health apps)*, with two and three subthemes, respectively aligning with the TFA. Translated excerpts are presented and contextualised, combining the chiropractors’ words with the analytic findings. In the results section, we present quotes that clearly and richly illustrate the themes rather than validate our findings [34]. Although some participants are more extensively represented due to their clarity of voice, similar perspectives were expressed by other participants across all cases, indicating thematic consistency. Pseudonyms are used throughout.

#### THEME 1. Aligning the perceived purpose of health apps with clinical practice values

The chiropractors generally categorised health apps into three distinct types; those containing patient health information, those providing information and advice directed at the patient, and those offering exercise instructions. This classification underscored the chiropractors’ perception of the multifaceted role that health apps can play in enhancing the quality of care by easy access to health information, while simultaneously emphasizing the need to critically evaluate the alignment of these tools with their practice.

##### 1.1 Streamlined information access for chiropractors (perceived effectiveness, affective attitude, and coherence)

The chiropractors viewed health apps as an easy and time-saving solution for obtaining patient health information, such as accessing medication records or looking up diagnostic imaging results.

So we can’t avoid using “MinSundhed”, because we often carry out scans [diagnostic imaging] where it takes too long to get the description, but by logging in with NemID [digital identification solution in Denmark] we can quickly find out roughly what it’s about, instead of waiting for something for 7 days. [Miles]

Since most patients always had a mobile phone with them, the information could potentially be readily available to the chiropractors. However, the chiropractors found that not all patients knew about or had the relevant apps installed on their phones.

No, no, there’s still quite a way to go before it becomes easily accessible. [Ben]

##### 1.2 Health apps: tools for information, advice, motivation, and exercise guidance (affective attitude and opportunity costs)

Chiropractors emphasized that to recommend a health app, it should be easy to access, user-friendly, and contain relevant information and advice tailored to each patient. Ideally, the apps should include exercise instructions, preferably for various body parts, not just for a single region. Reminders for training within the app were considered important, and for some, positive feedback from patients made them recommend an app more often.

… if I quickly found out that it was easy and also got positive feedback from my patients like ‘oh, that really helped me remember,’ well then I think I would use it more quickly. [Michael]

Chiropractors generally saw their role as promoting self-management and motivating patients to exercise, viewing health apps as a support tool. Some chiropractors believed that health apps could take over part of the task of informing the patients and providing exercise instructions, ideally through an AI-based app that adjusts the information and exercises based on patient progress, to avoid becoming just an electronic training sheet. However, most chiropractors still wanted to maintain personal contact with their patients and expressed concern about diminished face-to-face contact if apps were used too much.

Well, my role is to support and encourage, and yes. So, I see myself having an active role in, well, teaching them to understand that self-management plays a huge role in this. [Anne]

But I think there are too many things that are just handled by apps. Then you lose some of the interaction between people. [Petra]

They saw it as positive that they and the patient could monitor progress through an app but acknowledged challenges in accessing and utilizing data without it becoming time-consuming and negatively impacting patients.

##### THEME 2. Chiropractors divided: knowledge, barriers, and attitudes shape the usage of health apps

The chiropractors were divided on how health apps fit into their practice, and their use of apps was influenced by prior knowledge, potential barriers, and patient characteristics. The chiropractors’ attitudes and patient assessments drove which patients were recommended to use an app, with those not using health apps struggling to see the need for change and the added value of health apps.

##### 2.1 How familiarity, confidence, and digital skills shape chiropractors’ app recommendations (Self-efficacy and ethicality)

The chiropractors described different levels of experience and approaches to using health apps with patients, but many had little or no knowledge of available health apps. Those who frequently recommended apps expressed confidence in their use, while others, who did not recommend apps, expressed a lack of understanding about the benefits and features of health apps, uncertainty about recommending them, or limited digital skills.

… I usually call myself an IT klutz [laughs]. Because it’s just, it’s an uphill battle when you didn’t grow up with it. [Harry]

Most chiropractors felt it was important to try the apps themselves and be familiar with their content before recommending them to patients. When they were familiar with the content, they found it easier to give patients specific details, explain its relevance, and guide the patients in case of technical or other problems. Conversely, they did not feel confident recommending apps they had not used themselves.

[...] I don’t have enough knowledge about them [apps] to say ‘I think you should use this’ because ... then I’m also endorsing it, somehow. Yes, and I find that it can be difficult when I don’t have more, [uh] insight and maybe also knowledge about what it [an app] specifically contains, and what it does. Yes. [Anne]

The excerpt also points to the importance of the apps’ content aligning with the values and principles the chiropractors follow in their practice. This alignment underpinned the chiropractors’ reasoning for the need to be familiar with the app content before endorsing it. Additionally, many of the chiropractors placed more importance on their personal assessment of the value of the app than on whether its content and effectiveness were evidence-based. Only a few believed that evidence-based content was important, while others saw it as a secondary benefit or selling point.

For me, it is important that I personally think that it [an app] has good content, and that I think it is relevant for the patient. More so than there is some scientific study showing that it benefits this type of patient. [Emma]

Yes, then it is much easier for me to tell the patients ‘it’s not something I’m making up. Look here, there is research to support this’. [Petra]

##### 2.2 Balancing time, costs, and implementation barriers (Burden)

The chiropractors believed digital solutions would become a more integrated part of future practice. Most felt that once they become more familiar with available apps, incorporating them into daily routines would be relatively easy. This was supported by the experiences of chiropractors who already used apps. However, those who rarely or never recommended health apps were content with their current way of practising and expressed concerns about implementing apps in their work with patients. Some considered apps as either in competition with existing in-house services, such as having a physiotherapist overseeing training, or as adding little value to current practice. Consequently, they did not perceive a need to adopt apps in their practice.

Well [uh phew], all beginnings are difficult, and when it’s something new that seems like a supplement to something you already know works, then I think it [the implementation] becomes difficult because, well then it’s an extra thing I have to remember, and it’s something I have to use extra resources on in the beginning. [Michael]

There was little agreement among the chiropractors on how much time was needed during consultations to introduce apps to patients. Some felt it was important to prioritize time for this within the consultation and already set aside extra time to guide patients in exercises. Conversely, others mentioned that due to time constraints, they occasionally had to postpone introducing an app to a later appointment. Several chiropractors also believed they should be compensated for the additional effort required to introduce apps, noting that a lack of financial incentives would remain a barrier to implementing apps into their practice.

Yes, it is time-consuming. So, one could argue, as it takes time away from your treatment, what you have allocated for treatment, then it is certainly a motivating factor, if you [uh], also profit from it, that’s for sure. [Petra]

For most of the chiropractors, data security was not a major concern when using apps in clinical practice, mostly because they did not think the apps would store sensitive patient information. They left any decisions regarding data sharing to the patients without discussing it with them. One chiropractor explained that being from a generation that generally did not question how their data was used, he felt he had little influence over data flow anyway. In contrast, other chiropractors felt responsible for understanding data security before recommending an app.

… I think one of the big issues is who has access to data? Who collects all this data, what is this data used for? Who is it sold to? [Anne]

The cost of the app and out-of-pocket expenses for the patients influenced whether chiropractors recommended health apps. Several chiropractors only recommended free or reasonably priced apps, although there was no agreement on what was considered “reasonable.” Two chiropractors felt that patients needed to pay for the app to take it seriously. For apps with a monthly subscription, one chiropractor emphasized the importance of clearly explaining what the patient signed up for and how to cancel the subscription. Another chiropractor noted that, while she had no trouble justifying the cost of her treatments, she struggled to convince patients to pay for a health app that she recommended.

I don’t think they should get anything for free, then they are not as motivated. [Petra]

Apps that cost also hampers me…, that they [the patients] have to… that I have to make them pay more than what they already pay for the treatment. [Petra]

##### 2.3 Selecting and matching patients with health apps (Perceived effectiveness)

The chiropractors rarely encountered patients asking for app recommendations. More often, patients requested exercise instructions, and chiropractors felt that most patients were unaware that health apps could provide such guidance.

Opinions varied on the relevance of health apps for different age groups of patients. The majority agreed it would be more natural for younger patients to use apps and less so for older patients, and only one did not regard age as a barrier to app use. Beyond age, several chiropractors emphasized the importance of motivating inactive patients to increase physical activity. They often tailored their app suggestion based on their assessment of patient preferences before suggesting an app.

Well, I would say it’s clearly the older generation, they might not be as hardcore. [Uh] So it is often them who end up with a piece of paper in their hands. [Uh] Yes, yes, it’s probably a bit of a generalization. [Ben]

[Um] You know who you can give a few extra tips, and where you just know it doesn’t matter. Yes. You shouldn’t say to the independent farmer [uh] ‘Hey John, I have an app for you.’ He would just shake his head and say, ‘have you lost it? [Emma]

###### Convergence of quantitative and qualitative findings

The integrated findings of the quantitative and qualitative findings are presented in Table 3 and led to the identification of three areas of complementarity:

**Table 3:** Joint display of the integrated results

Sub-themes	Quantitative findings	Qualitative findings	Comparison and contrasts	
**THEME 1: Aligning the perceived purpose of health apps with clinical practice values**				
1.1 Streamlined information access for chiropractors	32.3% of the chiropractors agreed that health apps could give chiropractors access to objective information on patients’ health, and 55.2% answered neither/nor	“ I primarily use’*MinSundhed*’, where I can quickly see patients’ imaging results and blood work or hospital journals. It saves me the trouble of ordering [the information]*.*” [Free text response] “ It’s difficult to answer as I haven’t integrated health apps into my treatment at all, but I will be more investigative in the future. However, I lack the experience to answer your questions adequately.” [Free text response]	*Convergence*: Various opinions on the benefits of health apps as sources of information on patients’ medical history. For some, an easy way to gain access to patients’ health information, but most were undecided on the matter mainly owing to lack of experience	Area 1 of complementarity
1.2 Health apps: Tools for information, advice, motivation, and exercise guidance	70.5% agreed that health apps could provide patients with information	“ … but that they get something to take home that they can use themselves and that they can continue to use even when they finish a treatment course with me. [Uh] And that can benefit them both mentally and physically.” [Emma] “… I think it has great value to link treatment to [uh] a degree of pain science and pain education… [Uh] So I think that, for me, that also carries almost as much weight, I would say, as the exercises that will be suggested do.” [Simon]	*Convergence*: There was a high level of agreement on the potential value for patients in getting health information via apps *Expansion*: The chiropractors described apps as potential tools for repeating or reinforcing information given during the consultations. Most often, this was described in the context of apps providing exercise instructions or monitoring progress	
	73.9% preferred apps that were specific to the patient’s issue The most recommended type of apps was exercise instructions (11.8%) 34.0,0% agreed that health apps would increase patient motivation	“… let’s say we think of the perfect chiropractor app, that [uh], I’m a neck patient so I click on the neck. Then there are four stretching exercises and four strengthening exercises I can start doing, which is pretty much what every patient would be able to start doing.” [Emma] “… if it is to be done sensibly and effectively with such exercise therapy, then it is necessary that it can [uh], provide some exercises that also progress over time. … it’s not just a handful/ten fixed exercises with the same repetitions and, and stuff like that.” [Simon] “… For some, it will probably also be a [uh], well, a motivator, the fact that you can see your own goals, and you can constantly monitor, and you can see that you got it done today and there is someone cheering you on.” [Anne]	*Convergence*: There was a high level of agreement on the need for apps to be specific to the patients’ needs, but a lower level of agreement on the effect on motivation *Expansion*: The chiropractors mainly addressed the need for tailoring and progression of exercises, and saw the potential for monitoring as a motivational factor	
	32.3% of the chiropractors agreed that health apps could aid better patient care, and 51.7% answered neither/nor	“… I think that there are at least some people who don’t want to, where it would be more of a stress factor for them to use an app, [uh] than to remember it themselves, for example. So in terms of stress, I think it could potentially have a negative effect.” [Michael]	*Convergence*: There was limited agreement on the benefits of apps on patient care and concerns regarding the impact on the patient-provider relationship *Expansion*: The interviews pointed towards increased screen time and increased stress on patients as potential negatives. Further, the chiropractors discussed the continued need for personal contact and the app as a add-on to care rather than a substitute for care	
	8.4% agreed that health apps detracted from the personal element in chiropractor-patient relationship, and 33.7% disagreed	“ So I would be fine with that, because it’s still a dynamic between me and the patient, so we still talk about how things are going, how do you think the exercises are going, is there anything that’s not working, or is there anything that the app doesn’t take into account, or, well, there’s still some sparring, I guess.” [Anne]		
**THEME 2: Chiropractors divided: Knowledge, barriers, and attitudes shape the usage of health apps**				
2.1 How familiarity, confidence, and digital skills shape chiropractors’ app recommendations	72.9% were interested in learning how to use apps with patients The most commonly preferred platform for learning about apps were webinars (63.2%)	“ Well, it could be a webinar then. Which has worked really well. I don’t think it would require much more than that, so you can sit and play with it [um] on the side and use the app yourself and see what it is.” [Simon] “ So, all these things take too much time, I simply don’t have time for everything I want to do. If I didn’t have patients, I would have time for all these things.” [John]	*Convergence*: There was high level of interest in learning more about the apps with webinars being the preferred form of learning platform *Expansion*: Some chiropractors expressed time as a barrier for participating in post-graduate education, while others preferred self-learning	Area 2 of Complementarity
	75.4% agreed that an app should be tested and quality assured before they would recommend it to patients 81.9% would need to test an app before recommending it to patients	“ Well, if I don’t know anything about an app or feel I know anything about it, I can’t recommend it, I need to know what I am passing on.” [Camilla] “ Yes, it matters to me [that the app is evidence based], very much so, and that it is based on the same, I think, values and basic principles that I myself have in the treatment and our work as chiropractors.” [Simon] “ No, it’s not that it’s not primary, but I think it’s great if, if they can show some kind of [uh] result.” [Ben] “… The more there is on the sales list [uh] for good arguments. Yeah, definitely. I wouldn’t recommend an app, even if it was evidence-based, if it’s really, [uh] hard to access and doesn’t seem logical and you can’t find your way around it, and because then you won’t use it.” [Camilla]	*Convergence*: There was a high level of agreement on the need for testing and ensuring the quality of an app before recommending it *Divergence*: Many chiropractors expressed the importance of app content aligning with their values and way of practicing rather than emphasizing evidence-based content or formal testing. User friendliness and the chiropractors’ own preferences were mentioned as important for endorsing an app. When referring to effect, the focus was mainly on first-hand experience and positive patient feed-back	

Areas of *convergence* between the two phases: describes the agreement between the two sets of findings

Areas of *divergence* between the two phases: describe when the quantitative and the qualitative findings demonstrate conflicting interpretations

Areas of *expansion* where qualitative sources help to expand the survey findings. The ‘expansion’ label also indicates instances where the qualitative and quantitative data addressed the same phenomenon but in a different and informative way

^*^Agreement is defined as those who have answered “agree” or “completely agree” in the survey

- *Area 1:* Regarding the alignment of the perceived purpose of health apps with clinical practice values (theme 1), we found that many chiropractors viewed health apps as tools for providing information and exercise instructions to patients, either aligning well with their usual practice routines or as a supplement to usual practice. However, for others, the apps appeared to conflict with their usual practice and, by extension, posed a potential threat to their model of care and livelihood, which seemed to impact their perception of the value of apps for patients and improved patient care.
- *Area 2:* Under the subtheme of how familiarity, confidence and digital skills shape chiropractors’ app recommendations (subtheme 2.1), we observed a generally low level of knowledge of and experience with using health apps as tools for managing patients in the Danish chiropractic profession. The chiropractors expressed a strong interest in receiving formal education on the subject. However, the use of health apps with patients seemed to be shaped by issues not easily addressed in a formal teaching program, such as firsthand experience with an app, personal preferences, and alignment of the app’s content with personal values. There was a consensus that applications should undergo testing and quality assurance before being recommended to patients. However, the chiropractors expressed a preference for this process to be informed by their own first-hand experience. They emphasised that the content of the application should align with their professional values as chiropractors, rather than being driven by evidence-based research.
- *Area 3:* Finally, under the subthemes of balancing time, costs and implementation barriers, and selecting and matching patients with health apps (subthemes 2.2 and 2.3), we identified that chiropractors believed the high efforts to familiarize themselves with app content and lack of time during consultations were barriers to app use. But it is possible that perceptions of apps as non- or low-value care options conflict with the chiropractors’ practice models, and that both patient and chiropractor expectations of care models were stronger drivers of non-use.

## Discussion

The results of this study provide insights into Danish chiropractors’ use of health apps in daily practice and their attitudes towards implementing and working with these apps. While chiropractors acknowledged that health apps are becoming an increasingly integral part of healthcare, their limited knowledge and a narrow scope of app use indicate a need for new learning and implementation initiatives. The attitudes towards using health apps were greatly divided and will likely influence the further implementation of digital health interventions in chiropractic practice.

We found that only one in six chiropractors used health apps weekly or monthly in their work with patients. This is fewer [7, 19] or at the same level [21] as other HCPs. Danish chiropractors primarily used health apps to provide patients with information and support for exercising, whereas other HCPs used health apps for administrative purposes, such as accessing patient information, communicating with colleagues and patients, scheduling appointments, or prescribing medication [19, 35]. These features are integrated into most Danish electronic patient record systems, making app formats less relevant in the Danish context. This may partly explain why apps are used less frequently by chiropractors in Denmark than in comparable international settings.

Generally, the chiropractors expressed a strong desire to maintain autonomy in planning patients’ care programs, extending this to being able to adapt or modify content delivered by apps. Consistent with findings from other HCPs [13, 20, 36], concerns about preserving authority in patient care when integrating digital tools have resulted in a reluctance to adopt and implement e-health technologies [37]. In extension, the concern for loss of the patient-provider relationship has been a recurrent theme in similar studies [13, 15, 18, 22]. For example, Györffy et al. found that while the use of online resources increased patient involvement and understanding, it also raised concerns among HCPs about misinformation and the potential for misdiagnosis in online consultations [15].

Unlike HPCs in international studies [11, 12, 16, 17, 20], some of the chiropractors emphasised accessibility, user friendliness, and their personal appraisal of the apps over data security and evidence-based content. Danish apps endorsed by the Danish Health Authorities work under a heavy security layer compliant with the European General Data Protection Regulation, which may partly explain why some chiropractors did not see data security as a barrier to use. However, we also observed a common misconception that the absence of personal data entry in an apps means that there are no security concerns.

The chiropractors disagreed on the burden of recommending health apps as part of their work, with opinions ranging from time-consuming to timesaving. They also differed on whether it was acceptable to impose out-of-pocket expenses on the patients to buy an app and on reimbursement for their services. These perceptions likely reflect the varying views on the effectiveness and added value of health apps, with sceptic chiropractors being unsure of the apps’ potential for better or faster results than usual care. Similar patterns regarding the time issue have been observed elsewhere [11], and the cost issue [7, 12, 15, 17, 21, 22, 38] has been discussed by many HCPs, and opinions on who should pay vary. However, reimbursement for providing patients with instruction on health apps has commonly been seen as a key facilitator for HCPs, who have emphasised the need for clear guidelines on reimbursement policies before integrating health apps into clinical practice [15, 17, 22, 38].

Often, the chiropractors took it upon themselves to decide if a patient could use an app, sometimes excluding older patients without consulting them. Many chiropractors viewed their older patients as less capable of using health apps, a belief also observed in other HCPs [14, 19, 22]. However, Bhattarai et. al. [38] found that older patients are becoming more familiar with digital tools and could benefit from health apps. This suggests HCPs should reconsider assumptions about older patients’ abilities and integrate supportive strategies to support health app adoption. Additionally, the chiropractors believed only patients who were already self-managing would be interested in using such apps, while Sarradon-Eck et al. found that HCPs also consider factors like income levels, digital literacy, and linguistic abilities when deciding if patients should be offered health apps [20].

### Strengths and limitations

We have used a mixed-methods study to evaluate Danish chiropractors’ knowledge and attitudes towards health apps. By using both quantitative and qualitative data, we investigated the extent of chiropractors’ knowledge and attitudes towards health apps but also gained in-depth knowledge of their attitudes, facilitators and barriers towards apps. The questionnaire used in the survey was developed based on previously used questionnaires in other countries and adapted to the Danish context, resulting in high content validity [39]. The questionnaire was pilot tested before it was sent to the chiropractors, which strengthened the face validity [39], but future iterations of the questions should ensure an even higher degree of face validity to minimize the extent of missing data. The response rate of 45% limits the generalizability of our findings. However, when comparing with response rates ranging from 21 to 61% in other similar surveys of healthcare professionals [7, 16, 19], our response rate is relatively high.

For the interviews, we were able to recruit chiropractors who differed in gender, age, years of experience, and had different experiences with the use of health apps and were thus able to ensure the representation of diverse perspectives on health apps in clinical practice. The interviews were rich in detail and exhaustive on the topic, which strengthens our findings.

We cannot rule out the possibility that social desirability bias impacted the results. In both the survey and interviews, chiropractors may have expressed more positive attitudes towards health apps because they believed this was what the research team expected. This bias may have been more pronounced in the interviews, as the participants were aware that MMS, who conducted the interview, had a dual role as both a part-time researcher and a practising chiropractor in primary care.

### Implications for practice

Limited knowledge about health apps and concerns over their relevance and value in clinical practice are key barriers to their adoption by chiropractors. This highlights the need for targeted promotion of evidence-based apps and educational initiatives adapted to the needs and preferences of chiropractors to expand their knowledge of digital health tools. This also includes offering health apps to a wider spectrum of patients after a conversation, as part of an overall, patient-centered approach, and offering support to empower patients to self-manage their condition better. For health apps to be successfully adopted in Danish chiropractic practices, it is paramount that chiropractors recognize their potential benefits. Therefore, educational initiatives should address value-based concerns regarding the integration of these tools into patient care and answer questions such as *“What’s in it for me?”* and *“How does this fit into my practice?”*. To fully overcome these organizational and financial barriers, institutional or policy changes may be needed, including ensuring financial incentives and providing clear guidance on any out-of-pocket costs for patients.

## Conclusion

This study highlights the complex interplay between chiropractors’ attitudes, experiences, and the perceived utility of health apps in clinical practice. While there is a clear recognition of the potential benefits of these digital tools, significant barriers remain that hinder their widespread adoption. Addressing these barriers will require a multifaceted approach, including targeted education and training, the development of user-friendly applications that align with chiropractors’ practice models, and strategies to enhance patient engagement with health technology. As the chiropractic profession continues to evolve in the digital age, embracing health apps as integral components of patient care may ultimately lead to improved outcomes and enhanced patient experiences. The findings of this study contribute valuable insights to the ongoing discourse on digital health integration in chiropractic practice, paving the way for future research and development in this area.

## Supplementary Information

Additional file1 (DOCX 34 kb)

## Data Availability

The datasets used in the current study are available from the corresponding author on reasonable request.

## Abbreviations

MSK: Musculoskeletal
mHealth: Mobile health
HCP: Health care provider
TFA: Theoretical framework of acceptability
