# Supplementary material for: From scepticism to integration: adoption of mobile health apps in Danish chiropractic practice—a mixed methods study
Source: Chiropr Man Therap. 2025 Oct 24;33:49. doi: 10.1186/s12998-025-00604-0 (PMC12553236; doi:10.1186/s12998-025-00604-0)
Supplement: Supplementary file 1 — Additional file1 (DOCX 34 kb) [file 12998_2025_604_MOESM1_ESM.docx]

**Additional file 1: Questionnaire**

**Information about the project and consent:**

The purpose of this study is to elucidate Danish chiropractors' attitudes towards and use of health apps as a treatment tool in their daily work with patients. The questions concern chiropractors' own use of health apps and the use of these as part of treatment for patients in primary practice.

Before you agree to participate in the project, you need to know what is involved:Participation is voluntary and you can withdraw from the project if you wish.No sensitive personal data is collected, only common demographic data such as age, gender and year of graduation. All information you provide in the project is treated confidentially. The information will only be disclosed to researchers and their students. Before publication, all information will be anonymized so that it does not appear from the results who has contributed to the project. You can request access to your answers at any time and receive a copy by contacting the Data Controller. Furthermore, you can object to the processing of the data or file a complaint with the Danish Data Protection Agency about the data processing. The data will only be stored in personally identifiable form for as long as it is necessary for the purposes of our research. Thereafter, it will be anonymized or deleted.
This consent form is the legal basis for us to use your data for research purposes.

Yours sincerely

Data Controller:
Emilie Søbeck Wittrock, emwit18@student.sdu.dk
Jeppe Nørbæk Laursen, jelau18@student.sdu.dk
Mette Tølbøl Johannesen, mejoh18@student.sdu.dk

Responsible supervisor: Senior Researcher Mette Jensen Stochkendahl, Chiropractors' Knowledge Center, m.jensen@kiroviden.sdu.dk

# 1. I hereby give my consent to participate in the study

(1) Yes

(2) No

(3) I do not see patients, so the project is not relevant to me.

# 2. Gender

(2) Female

(1) Male

(3) Other gender identity

# 3. Age

(1) 21-30 years

(2) 31-40 years

(3) 41-50 years

(4) 51-60 years

(5) 61-70 years

(6) >70 years

# 4. Years since graduation?

(1) 0-5 years

(2) 6-10 years

(3) 11-15 years

(4) 16-20 years

(5) >21 years

# 5. What employment(s) do you have?

(1) Clinic owner

(2) Employee in clinic

(3) Intern

(4) Teacher

(5) Researcher

(6) Public employee

(7) Working at health insurance

(8) Other employment

# 6. In which region(s) do you work?

(1) Capital Region

(2) Zealand

(3) Southern Denmark

(4) Mid Jutland

(5) North Jutland

# 7. How many patients do you see per hour on average?

(1) 1-2

(2) 3-4

(3) 5-6

(4) >6

**The following questions address your attitude towards and use of health apps as a treatment tool in your daily work with patients.**
Definition of a health app:
A health app is an application program that offers health-related content to users of smartphones and tablets. Health apps allow patients to collect, record and monitor information about their health, such as training, exercise and sleep. Through health apps, it is also possible to deliver targeted patient information about diseases and conditions.
A health app can be offered by the Danish healthcare system (e.g. MinLæge) or by a commercial provider (e.g. Garmin Connect).

# 8. Do you use health apps for personal use?

(1) Yes

(2) No

# 8.1. What categories of health apps do you use for personal use?

(1) Training/exercises

(2) Fitness tracking (e.g. step counts)

(3) Apps from the Danish health authority (e.g. “MinSundhed”)

(4) Sleep

(5) Nutrition and weight

(6) Smoking cessation

(7) Menstrual cycle

(8) Stress and mental health

(9) Other _____

# 9. Do you use health apps in your daily work with patients?

(1) Yes

(2) No

# 9.1 Which categories of health apps have you recommended to your patients?

(1) Training/exercises

(2) Fitness tracking (e.g. step counts)

(3) Apps from the Danish health authority (e.g. “MinSundhed”)

(4) Sleep

(5) Nutrition and weight

(6) Smoking cessation

(7) Menstrual cycle

(8) Stress and mental health

(9) Other _____

# 9.2 How often do you recommend the use of health apps to your patients?

(1) Daily

(2) Weekly

(3) Monthly

(4) Rarely

# 9.3 Which health apps have you used in your daily work with patients?

(1) MyFitnessPal

(2) Exor Live

(24) Exercise Pain Away (TrænSmertenVæk)

(3) Garmin Connect

(16) Built-in health app on phone

(23) Selfback

(17) Daylio Diary

(18) Pain Diary

(19) My Headache (“Min Hovedpine”)

(20) Headspace

(21) MyDoctor (“MinLæge”)

(22) MyHealth (“MinSundhed”)

(25) The Medicine Card (“Medicinkortet”)

(15) Other _____

# 10. How much do you agree with the following statements?

|  | Strongly disagree | Disagree | Neither/ nor | Agree | Strongly agree |
| --- | --- | --- | --- | --- | --- |
| Health apps can help provide my patients with information about health and disease. | (1) 🔾 | (2) 🔾 | (3) 🔾 | (4) 🔾 | (5) 🔾 |
| Data from health apps can help me provide better care to my patients. | (1) 🔾 | (2) 🔾 | (3) 🔾 | (4) 🔾 | (5) 🔾 |
| Health apps gives me objective information about my patients’ health. | (1) 🔾 | (2) 🔾 | (3) 🔾 | (4) 🔾 | (5) 🔾 |
| Health apps allow me to meet my patients in their own homes. | (1) 🔾 | (2) 🔾 | (3) 🔾 | (4) 🔾 | (5) 🔾 |
| Health apps can contribute to better collaboration with other health care providers involved in my patients’ care. | (1) 🔾 | (2) 🔾 | (3) 🔾 | (4) 🔾 | (5) 🔾 |

# 11. How much do you agree with the following statements?

|  | Strongly disagree | Disagree | Neither/ nor | Agree | Strongly agree |
| --- | --- | --- | --- | --- | --- |
| The use of health apps means more work for me. | (1) 🔾 | (2) 🔾 | (3) 🔾 | (4) 🔾 | (5) 🔾 |
| Health apps are too time-consuming for me to use with my patients. | (1) 🔾 | (2) 🔾 | (3) 🔾 | (4) 🔾 | (5) 🔾 |
| Health apps detract from the personal element of the relationship between my patients and me. | (1) 🔾 | (2) 🔾 | (3) 🔾 | (4) 🔾 | (5) 🔾 |
| Health apps generate too much data, which can create a barrier to fast and efficient treatment of my patients. | (1) 🔾 | (2) 🔾 | (3) 🔾 | (4) 🔾 | (5) 🔾 |
| I am concerned about my patients’ data from health apps being used for commercial purposes. | (1) 🔾 | (2) 🔾 | (3) 🔾 | (4) 🔾 | (5) 🔾 |

# 12. How much do you agree with the following statements?

|  | Strongly disagree | Disagree | Neither/ nor | Agree | Strongly agree |
| --- | --- | --- | --- | --- | --- |
| My patients are interested in using health apps. | (1) 🔾 | (2) 🔾 | (3) 🔾 | (4) 🔾 | (5) 🔾 |
| Health apps are easy to use for my patients. | (1) 🔾 | (2) 🔾 | (3) 🔾 | (4) 🔾 | (5) 🔾 |
| Health apps raise motivation and willingness in my patients to manage their own health. | (1) 🔾 | (2) 🔾 | (3) 🔾 | (4) 🔾 | (5) 🔾 |
| It is an advantage if my patients can be recommended health apps that are specific to their issue. | (1) 🔾 | (2) 🔾 | (3) 🔾 | (4) 🔾 | (5) 🔾 |

# 13. How much do you agree with the following statements?

|  | Strongly disagree | Disagree | Neither/ nor | Agree | Strongly agree |
| --- | --- | --- | --- | --- | --- |
| I have enough knowledge about health apps to decide whether to recommend them to my patients. | (1) 🔾 | (2) 🔾 | (3) 🔾 | (4) 🔾 | (5) 🔾 |
| I need to test a health app before I recommend it to my patients. | (1) 🔾 | (2) 🔾 | (3) 🔾 | (4) 🔾 | (5) 🔾 |
| Health apps needs to be quality assured before I will recommend them to my patients. | (1) 🔾 | (2) 🔾 | (3) 🔾 | (4) 🔾 | (5) 🔾 |

# 14. Would you like to learn more about the use of health apps in your daily work with patients?

(1) Yes

(2) No

# 14.1 How would you like to receive training on the use of health apps?

(1) Webinar

(2) Illustrations

(3) Podcast

(4) Lectures

(5) Written/online information

(6) Courses

(7) Other _____

# 15. Do you have any further comments on the use of health apps in relation to your daily work with patients?

_____

# 16. This survey will be supplemented with interviews about chiropractors' use of health apps. May we contact you for an interview?

(1) Yes

(2) No

# 16.1 Please fill in your contact details so we have the opportunity to contact you

| Name | ________________________________________ ________________________________________ |
| --- | --- |
| Phone number | ________________________________________ ________________________________________ |
| E-mail | ________________________________________ ________________________________________ |

Thank you for participating in our survey.
 To finish the survey, please click "Finish" below.

**Additional file 2: Interview guide**

| Opening questions  Can we start by telling me why you have chosen this profession?  What is the best part about being a chiropractor?  What kind of issues do people you see in your clinic present with most often? Can you share something about your experiences of treating people with musculoskeletal conditions?  What do you think about the role of chiropractors in promoting/supporting self-management? |
| --- |
| Introduction to digital health tools  Today ‘s interview will focus on digital health tools. Digital health is an umbrella term that includes mobile health (mHealth) apps, electronic health records (EHRs), wearable devices, telehealth, and telemedicine. I would like us to focus specifically on mHealth apps and wearables if that is ok with you. |
| Core questions  Do you use health apps/wearables yourself?  If the answer is no- why not?  If the answer is yes- Which ones? How frequently? |
| What do you think about prescribing/recommending health apps to people who live with MSK conditions? Could you provide an example of when you recommended an app/platform to a patient? |
| If you haven’t recommended health apps at all/ only rarely – What are the reasons for that? What would help you to recommend health apps to patients (or what will help you do it more often?) |
| What are your thoughts on receiving training in the use of digital tools, (webinars, courses, podcasts)? |
| How good is your understanding /knowledge of digital health tools and how to use them? How about your patients’ level of EHealth literacy? |
| In your opinion, how effective are digital tools in helping people self-manage? |
| What do you think about the potential benefits of using these digital tools? (Giving patients faster access to providers and care, the possibility for remote monitoring, support with lifestyle change, adherence to treatment, and psychological support) |
| How important is it for you that digital tools are evidence-based? |
| What do you think are the potential drawbacks of using these tools? (Fewer customers/ less income, no financial incentive to recommend them/time consuming, loss of personal interaction with patients...) |
| How much effort do you think it would take to implement digital health tools in your practice? |
| Task  Rank the most important barriers to recommending digital tools to people with MSK  1,2,3,4 – Can add other barriers   - Lack of knowledge of effective apps - Concerns about data safety and security - Limited consultation time - Lack of training/guidance on implementation - A potential fee for the digital tool   Follow-up on rankings  Can you tell me more about why you ranked… as most important? |
| Summary of topics covered in the interview  Do you want to add something else? |

**Additional file 3: The qualitative analytic process**

| Analytic step | Step 1  Familiarisation | Step 2  Identifying an initial thematic framework | Step 3  Indexing and sorting | Step 4  Framework summarization | Step 5  Abstraction and interpretation |
| --- | --- | --- | --- | --- | --- |
| Analytic process | Familiarising with data and identify preliminary themes | Identify and sort topics into themes and subthemes | Labelling data according to the thematic framework | Condensation and summarization of data | Synthesising the findings and offer explicit and implicit explanations of findings. |
| Performing the analysis | MMS transcribed the interviews, reviewed the taped interviews, and read the transcripts to identify preliminary themes. | MMS reread the text and sorted the preliminary themes into meaning units based on the theoretical framework of acceptability. Any opposing units outside of the framework were included to cover all preliminary themes. | MMS carefully reread the text and coded the data according to the thematic framework, identifying various themes and subthemes. After the initial coding, all codes were reviewed, and some were merged while others were recoded into other codes. | MMS compiled all quotes within each theme and conducted a summary of each participants’ views within each theme or subtheme. | MMS reviewed all resumes for each theme and subtheme and began to group quotes into new themes based on these. This resulted in 8 different themes. MMS and MJS revised the themes and reached consensus, resulting in 2 themes and 5 subthemes. |
| Analytic tools used | From audio to text | By hand and Nvivo | Nvivo | Nvivo, excel and Word | Word and by hand |
| Units of analysis | 16 preliminary themes | 15 themes | 26 codes | 26 themes | 2 themes and 5 subthemes |
